# NPM2 in malignant peritoneal mesothelioma: from basic tumor biology to clinical medicine

**DOI:** 10.1186/s12957-022-02604-3

**Published:** 2022-04-30

**Authors:** He-liang Wu, Zhi-ran Yang, Li-jun Yan, Yan-dong Su, Ru Ma, Yan Li

**Affiliations:** 1 Department of Peritoneal Cancer Surgery, Beijing Shijitan Hospital, Peking University Ninth School of Clinical Medicine, No. 10 Tieyi Road, Yangfangdian Street, Haidian District, Beijing, 100038 China; 2 Department of Peritoneal Cancer Surgery, Beijing Shijitan Hospital, Capital Medical University, Beijing, China

**Keywords:** Nucleoplasmin2, Gene structure, Malignant peritoneal mesothelioma, Molecular targeting therapy

## Abstract

**Background:**

This review systematically summarizes gene biology features and protein structure of nucleoplasmin2 (NPM2) and the relationship between NPM2 and malignant peritoneal mesothelioma (MPM), in order to explore the molecular pathological mechanism of MPM and explore new therapeutic targets.

**Methods:**

NCBI PubMed database was used for the literature search. NCBI Gene and Protein databases, Ensembl Genome Browser, UniProt, and RCSB PDB database were used for gene and protein review. Three online tools (Consurf, DoGSiteScorer, and ZdockServer), the GEPIA database, and the Cancer Genome Atlas were used to analyze bioinformatics characteristics for NPM2 protein.

**Results:**

The main structural domains of NPM2 protein include the N-terminal core region, acidic region, and motif and disordered region. The N-terminal core region, involved in histone binding, is the most conserved domain in the nucleoplasmin (NPM) family. NPM2 with a large acidic tract in its C-terminal tail (NPM2-A2) is able to bind histones and form large complexes. Bioinformatics results indicated that NPM2 expression was correlated with the pathology of multiple tumors. Among mesothelioma patients, 5-year survival of patients with low-NPM2-expression was significantly higher than that of the high-NPM2-expression patients. NPM2 can facilitate the formation of histone deacetylation. NPM2 may promote histone deacetylation and inhibit the related-gene transcription, thus leading to abnormal proliferation, invasion, and metastasis of MPM.

**Conclusion:**

NPM2 may play a key role in the development and progression of MPM.

## Introduction

Malignant peritoneal mesothelioma (MPM) is a rare malignant tumor arising from peritoneal mesothelioma, accounting for 7–30% of all malignant mesothelioma [1]. The World Health Organization (WHO) classifies the histological types of MPM into three major types, epithelioid, sarcomatoid, and biphasic, with epithelioid being the most common [2]. MPM, with high malignant potential and unclear pathogenesis, has a high mortality rate and extremely poor prognosis. There is an urgent need to study the molecular pathogenesis of MPM and explore new therapeutic targets.

**Fig. 1:**
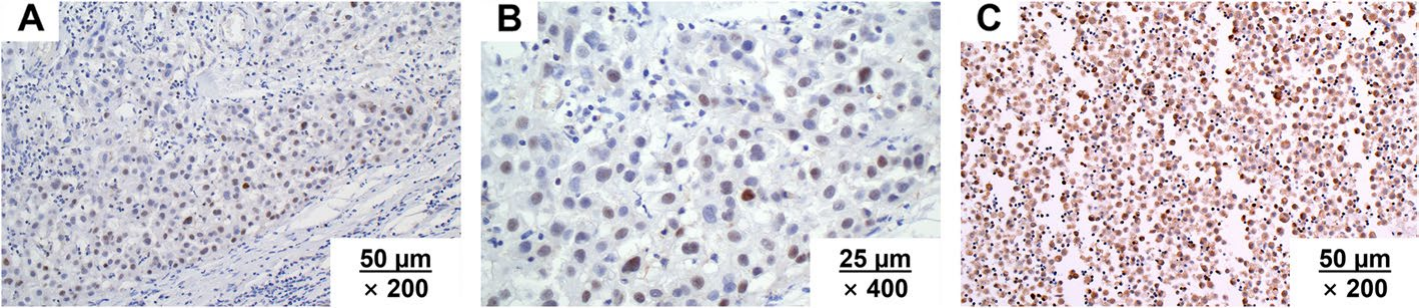
The pathological manifestations of MPM stained by NPM2 (IHC, catalog number ab243544). **A**, **B** NPM2 staining was positive in MPM patients (**A** ×200; **B** ×400). **C** Patient-derived tumor-like cell cluster result showed NPM2 staining was strongly positive in MPM (**C** ×200). Unpublished data from the authors’ group

In the public databases (GEPIA database and the Cancer Genome Atlas), our group found that NPM2 expression was correlated with the pathology of multiple tumors. Among mesothelioma patients, 5-year survival of patients with low-NPM2-expression was significantly higher than that of the high-NPM2-expression patients. Furthermore, NPM2 staining was positive in MPM by immunohistochemistry (IHC), suggesting NPM2 may be a potential therapeutic target for MPM (Fig. 1). This review is to summarize the gene biological characteristics and protein structure of NPM2, as well as its correlation with MPM.

## Biological characteristics of NPM2

### NPM2 gene structure

**Fig. 2:**
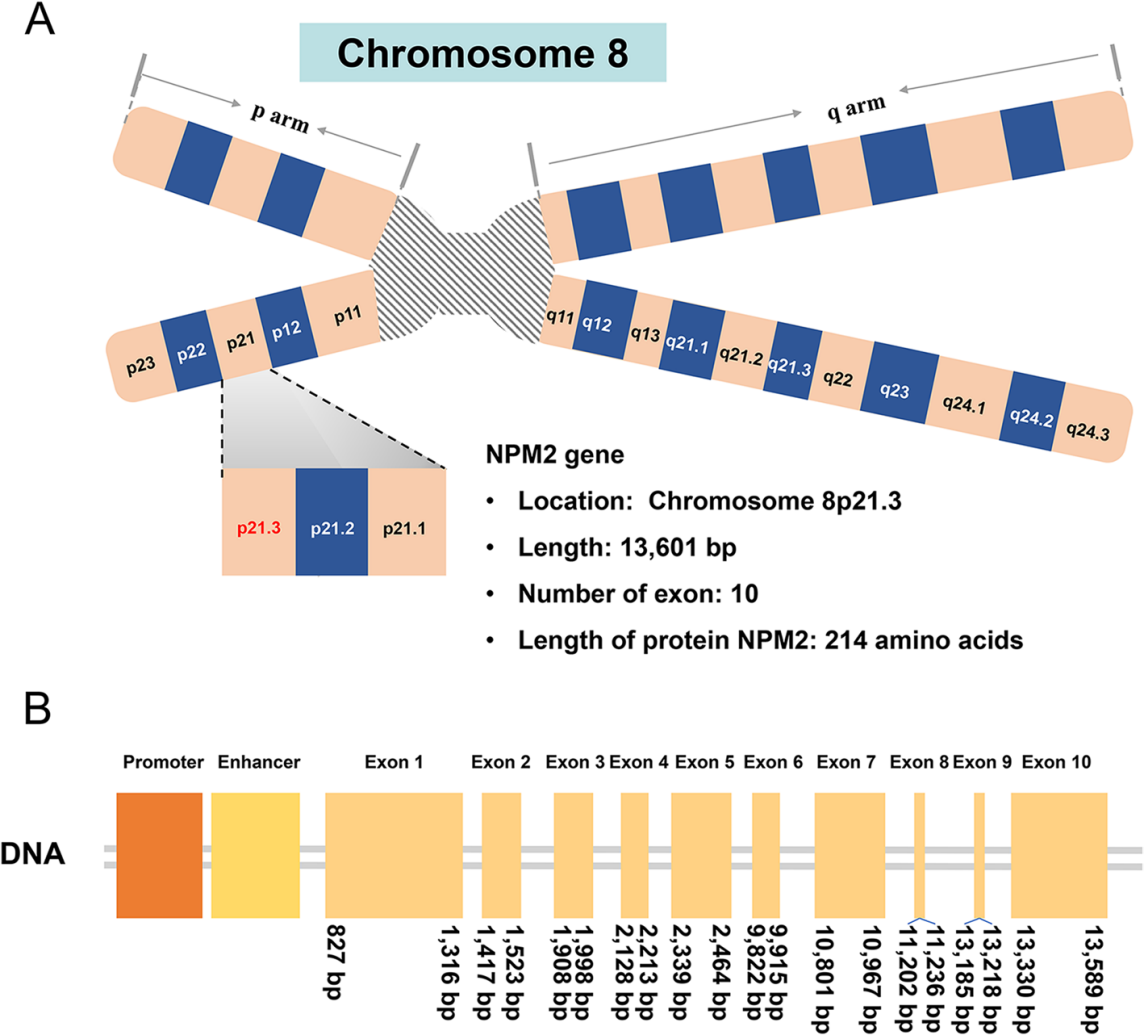
Schematic diagram of NPM2 gene. NPM2 gene is located on chromosome 8p21.3, with 10 exons

The NMP2 gene is located on chromosome 8p21.3, with a total base pair length of 13,601 bp, including 10 coding exons and 9 introns, encoding the NPM2 protein (Fig. 2) (Ensembl: ENSG00000158806 MIM:608073).

### NPM2 protein structure

**Fig. 3:**
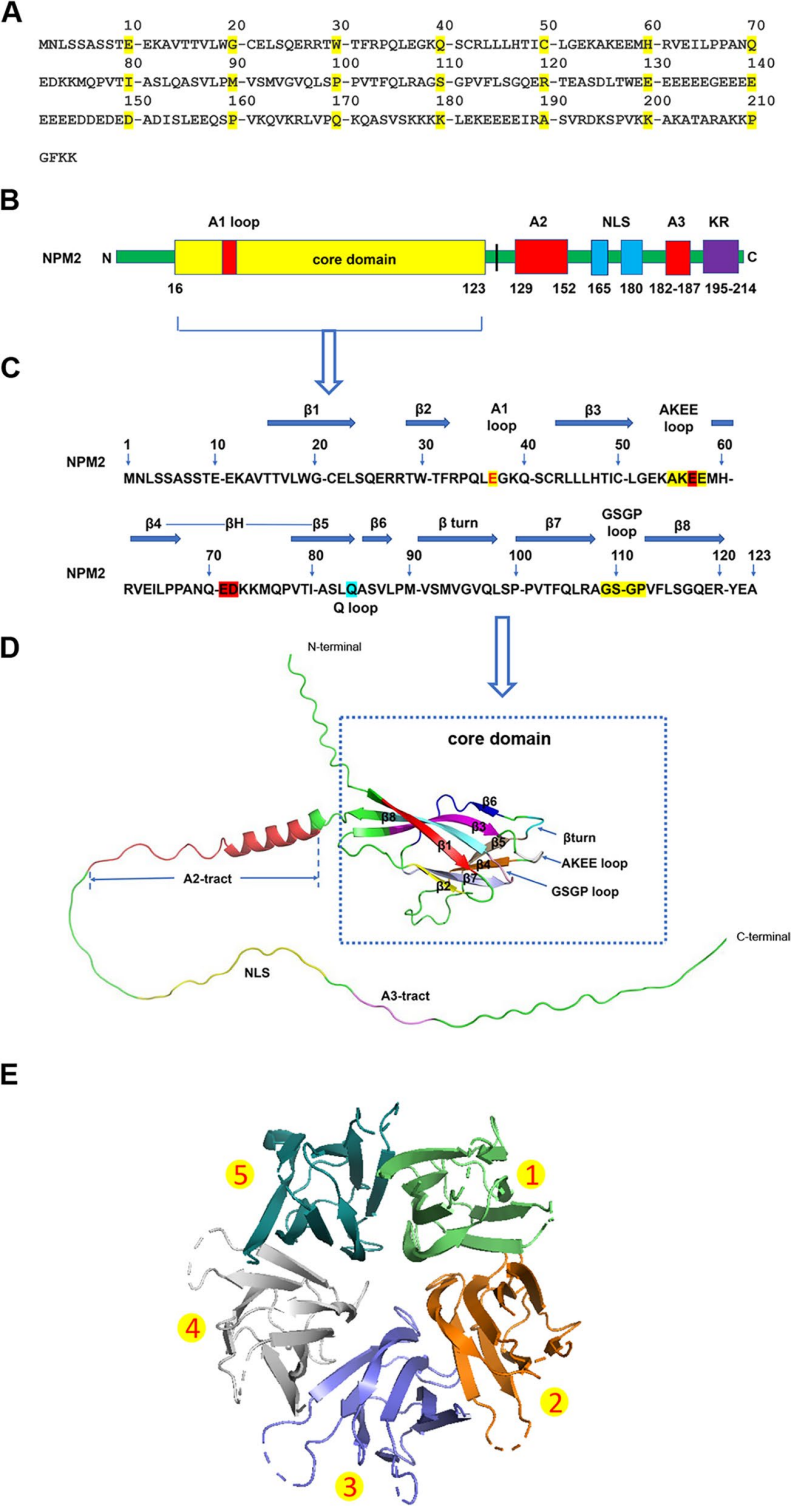
Schematic diagram of the NPM2 protein structure. **A** NPM2 amino acid sequence. **B** The major domain of NPM2 protein. The yellow, red, blue, and purple areas represent the core region, the acidic region, the nuclear localization signal, and the basic KR-rich region, respectively. **C** Secondary structure of N-terminal core region of NMP2 protein. The yellow, red, and blue represent important loops, vital acidic amino acid residues, and participation in decamer formation, respectively. **D** The prediction model of NPM2 protein. **E** The pentamer structure of NPM2 protein

The full-length NPM2 protein contains 214 amino acids (Fig. 3A). The main structural domains of NPM2 protein include the N-terminal core region, acidic region, and motif and disordered region (Fig. 3B). The protein structure is complex and the functional NPM2 structure contains pentamers (Fig. 3C–E) or decamers with 5 or 10 subunits (https://www.uniprot.org/uniprot/Q86SE8).

#### N-terminal core region

The N-terminal core region, located at amino acids 14–122, is connected by β strand, β turn, and loop in the following manner: β1→β2→A1 loop→β3→AKEE loop→β4→ βH→β5→Q loop→β turn→β6→β7→GSGP loop→β8 (Table 1, Fig. 3C, D). The members of the nucleoplasmin (NPM) family have similar amino acid sequences, major domains, and tertiary/quaternary structures [3, 4]. The N-terminal core region of NPM2 has many special structures: (1) NPM2 has a special βH structure, located at amino acids 63–80. The βH residues in NPM2 subunit are 3 more than that of *Xenopus* nucleoplasmin. The amino acid residues 68–76 are disordered. (2) NPM2 contains an A1 loop located between β2 and β3, which is a single acidic residue (Glu37) instead of multiple disordered acidic residues (3–12). (3) The bulge of the AKEE loop may be involved in the formation of decamers. (4) The salt bridge network is anchored by Lys56 and Glu58 in the AKEE loop, while Ser110, Gln25, Glu26, and Ser24 are involved in the formation. (5) Glu57 in the AKEE loop forms a hydrogen bond with Ser86 in the adjacent Q loop. (6) The highly conserved glutamic acid (Glu63) on βH forms hydrogen bonds with Gln105, Arg61, and Thr79. βH may be distorted due to the presence of consecutive proline residues (Pro66, Pro67). Since they flank two pentamers, these flexible βH can play an important role in histone binding [4].

**Table 1:** Specific secondary structure location of the N-terminal core region of NPM2 protein

Feature key	Position(s)
β1	16–23
β2	29–32
A1 loop	37
β3	43–51
AKEE loop	55–58
β4	60–65
βH	63–80
β5	78–84
Q loop	84
βturn	85–87
β6	90–98
β7	100–109
GSGP loop	109–112
β8	113–120

#### Acidic region

NPM2 contains three acidic regions, namely NPM2-A1, NPM2-A2, and NPM2-A3. All the N-terminal core regions of the NPM family contain a short A1 acidic region, known as A1-tract. However, NPM2-A1 is a single acidic amino acid (Glu37), known as A1-loop. NPM2 pentamers lack the typical A1 acidic domain, a property that may account for the inability of the N-terminal core region of NPM2 to bind histones directly. NPM2-A2 is the longest acidic region in the NPM2 protein, known as A2-tract, located at amino acids 129–152, containing 22 acidic amino acids. NPM2-A2 plays the most important role in binding NPM2 to histones [4]. NPM2-A3, located at amino acids 182–187, may play an auxiliary role in binding to histones.

#### Motif

NPM2 contains a motif located at amino acids 165–180, which is a classical nuclear localization signal (NLS). NLS is a short peptide sequence that exists on many proteins. It can specifically bind to nucleoplasmic transporter receptors to mediate the active transport of cargo proteins through nuclear pore complexes into the nucleus, thus enabling the replication of cargo proteins in the nucleus. NLS has diverse forms and different mechanisms and plays a significant role in the normal physiological functions of nucleoproteins [5–8].

#### Disordered region

NPM2 contains many disordered sequences, mainly concentrated in amino acids 119–214. Researchers have discovered a new type of proteins that have uncertain three-dimensional structure and specific biological functions. All or part of the regions of such proteins, which cannot themselves fold into a definite and unique spatial structure, are disordered regions. When combined with other proteins, they can fold into specific structures, so there are a series of rapidly interconverting conformations. Disordered proteins have crucial functions in many aspects, including regulation of transcription, translation, cell signaling, protein phosphorylation, storage of small molecules, and regulation of self-assembly of large multiprotein complexes [9, 10].

## Bioinformatics characteristics of NPM2

### Conserved sequence

**Fig. 4:**
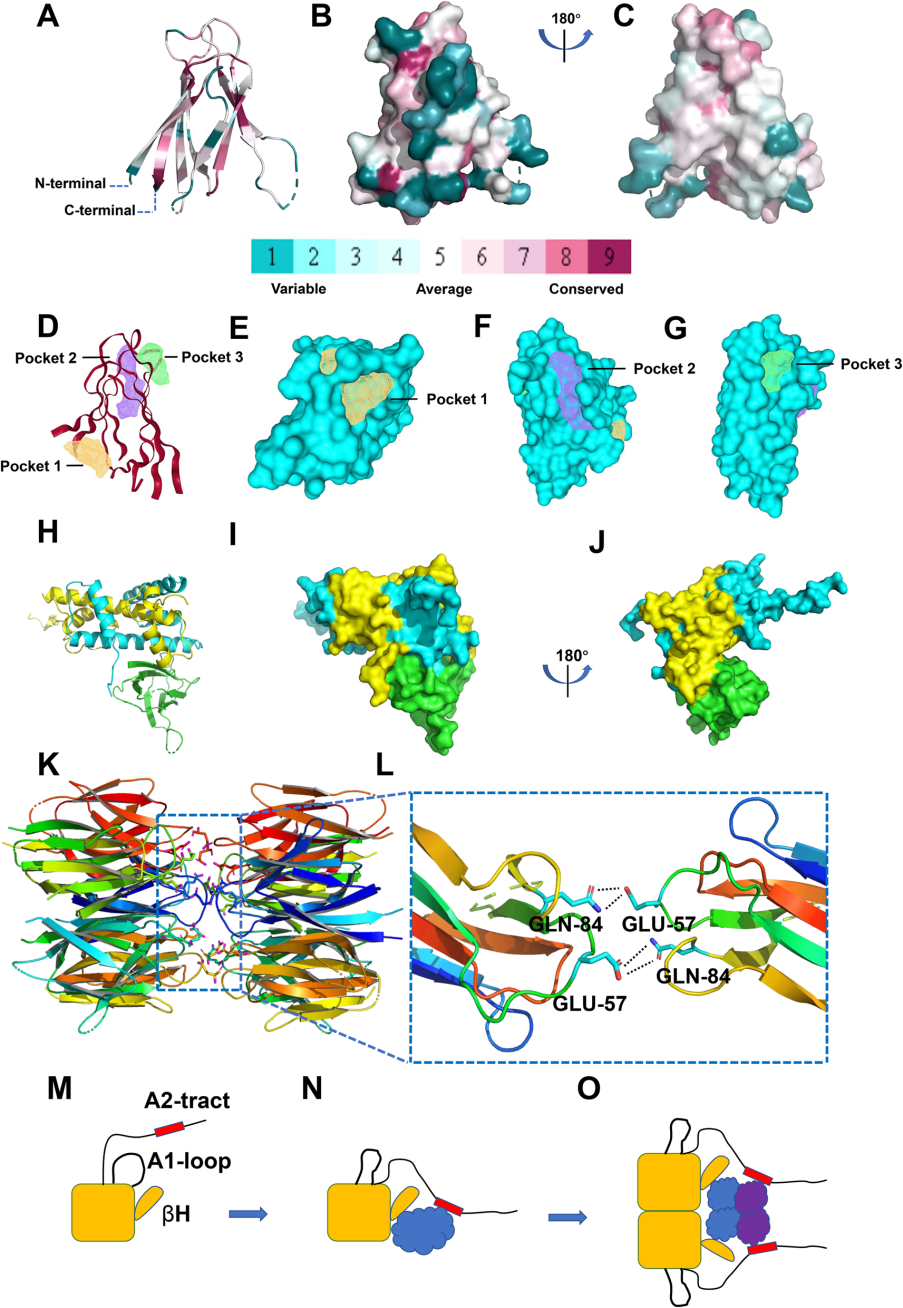
Bioinformatics characteristics of NPM2 protein. **A**–**C** Homology comparison of NPM2-core amino acid sequences. **A** The homology comparison result is shown as ribbons. The red, white, and blue areas represent conserved, average, and variable sequences of NPM2 protein, respectively. **B**, **C** The homology comparison result is shown as surfaces. **D**–**G** Predicted binding pockets of NPM2-core. **D** The result of predicted binding pockets is shown as ribbons. **E**–**G** The results of predicted binding pockets 1–3 are shown as surfaces. **H**–**J** Prediction diagram of NPM2 binding to histone H2A-H2B dimers. ZdockServer online tool (https://zdock.umassmed.edu/) was used to predict the binding between NPM2 protein and histone H2A-H2B dimers. **H** The prediction result is shown as ribbons. The blue, yellow, and green areas represent histone H2A, histone H2B, and NPM2, respectively. **I**, **J** The prediction result is shown as surfaces. **K**, **L** A decamer model for NPM2-core. **K** A side view of a modeled NPM2-core decamer is shown as ribbons. Side chains of Glu57 and Gln84 within the interface are shown as sticks. **L** The side chain of Glu57 in the AKEE loop would allow the formation of hydrogen bonds between Glu57 and Gln84 in opposing subunits in the modeled NPM2 decamer. **M**–**O** Side view of NPM2 pentamer/decamer binding to histones. **M** The structure of NPM2 pentamer. The yellow area is the core region of the NPM2 pentamer, and the red area represents the acidic region of NPM2-A2. **N** NPM2 pentamer binds to histone H2A-H2B dimers. The blue area represents the H2A-H2B dimers. **O** NPM2 decamer binds a mixture of H2A-H2B dimers and H3-H4 tetramers to form histone octamers on the chaperone. The purple area represents the H3-H4 tetramer

Consurf online tool (https://consurf.tau.ac.il/) was applied to detect conserved sequences of NPM2 protein, which plays the most important roles in maintaining protein structure and function (Fig. 4A–C). The N-terminal core region, involved in histone binding, is the most conserved domain in the NPM family and mainly responsible for the formation of pentamers and decamers [11–14].

### Binding pocket

DoGSiteScorer online tool (https://proteins.plus/) was applied to predict and describe potential binding pockets. DoGSiteScorer tool can detect potential binding pockets within a specific protein and then rank these pockets according to their size, surface area, and druggability score. NPM2 protein has 3 binding pockets, which may be potential sites for histone binding (Fig. 4D–G).

### The binding of NPM2 to histones

There are specific structures that bind NPM2 to histones: NPM2-A2 acidic region, βH structure, and disordered region. NPM2 may first bind to histone H2A-H2B dimers (Fig. 4H–J). E57 in the pentameric AKEE loop forms hydrogen bonds with Q84 in the opposite subunit (Fig. 4K, L). Upon dimerization of histone H3-H4 tetramers, two NPM2 pentamers form a compact decamer. Histones H2A-H2B and H3-H4 form octamers and bind to NPM2 decamers [4]. During the binding of NPM2 pentamers/decamers to histones, disordered regions may rapidly change conformation, fold into ordered sequences, and assist other specific structures to bind to histone dimers/tetramers/octamers (Fig. 4M–O).

### The diagnostic significance of NPM2 in human cancers

**Fig. 5:**
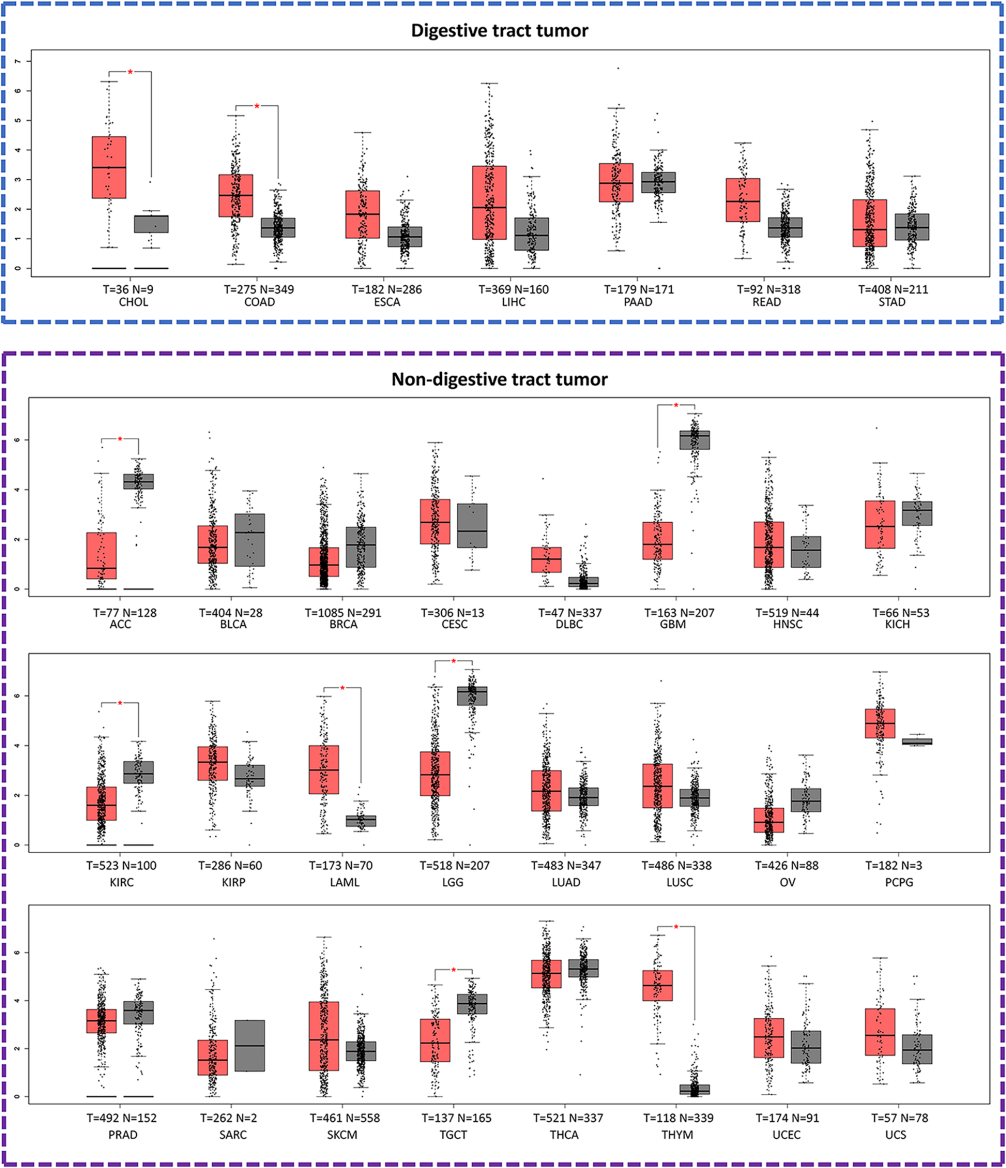
The expression of NPM2 in different tumors (*P<0.01). T, tumor tissue; N, normal tissue; CHOL, cholangiocarcinoma; COAD, colon adenocarcinoma; ESCA, esophageal carcinoma; LIHC, liver hepatocellular carcinoma; PAAD, pancreatic adenocarcinoma; READ, rectum adenocarcinoma; STAD, stomach adenocarcinoma; ACC, adrenocortical carcinoma; BLCA, bladder urothelial carcinoma; BRCA, breast invasive carcinoma; CESC, cervical squamous cell carcinoma and endocervical adenocarcinoma; DLBC, lymphoid neoplasm diffuse large B-cell lymphoma; GBM, glioblastoma multiforme; HNSC, head and neck squamous cell carcinoma; KICH, kidney chromophobe; KIRC, kidney chromophobe; KIRP, kidney renal papillary cell carcinoma; LAML, acute myeloid leukemia; LGG, brain lower grade glioma; LUAD, lung adenocarcinoma; LUSC, lung squamous cell carcinoma; OV, ovarian serous cystadenocarcinoma; PCPG, pheochromocytoma and paraganglioma; PRAD, prostate adenocarcinoma; SARC, sarcoma; SKCM, skin cutaneous melanoma; TGCT, testicular germ cell tumors; THYM, thymoma; UCEC, uterine corpus endometrial carcinoma; UCS, uterine carcinosarcoma

In order to study the expression of NPM2 in various tumors and the correlation between NPM2 and diagnosis of patients, we input the gene name NPM2 into the GEPIA database (http://gepia.cancer-pku.cn/) and used a box plot to show the expression level of NPM2 in multiple tumors. Wilcoxon assay was applied to calculate the expression difference between normal tissues and tumor tissues. It was found that the expression of NPM2 was significantly different in multiple tumors: in digestive tract tumors such as cholangiocarcinoma (CHOL) and colon adenocarcinoma (COAD) and in non-digestive tract tumors such as adrenocortical carcinoma (ACC), glioblastoma multiforme (GBM), kidney renal clear cell carcinoma (KIRC), acute myeloid leukemia (LAML), brain low-grade glioma (LGG), testicular germ cell tumors (TGCT), and thymoma (THYM) (Fig. 5).

### The prognostic significance of NPM2 in mesothelioma

**Fig. 6:**
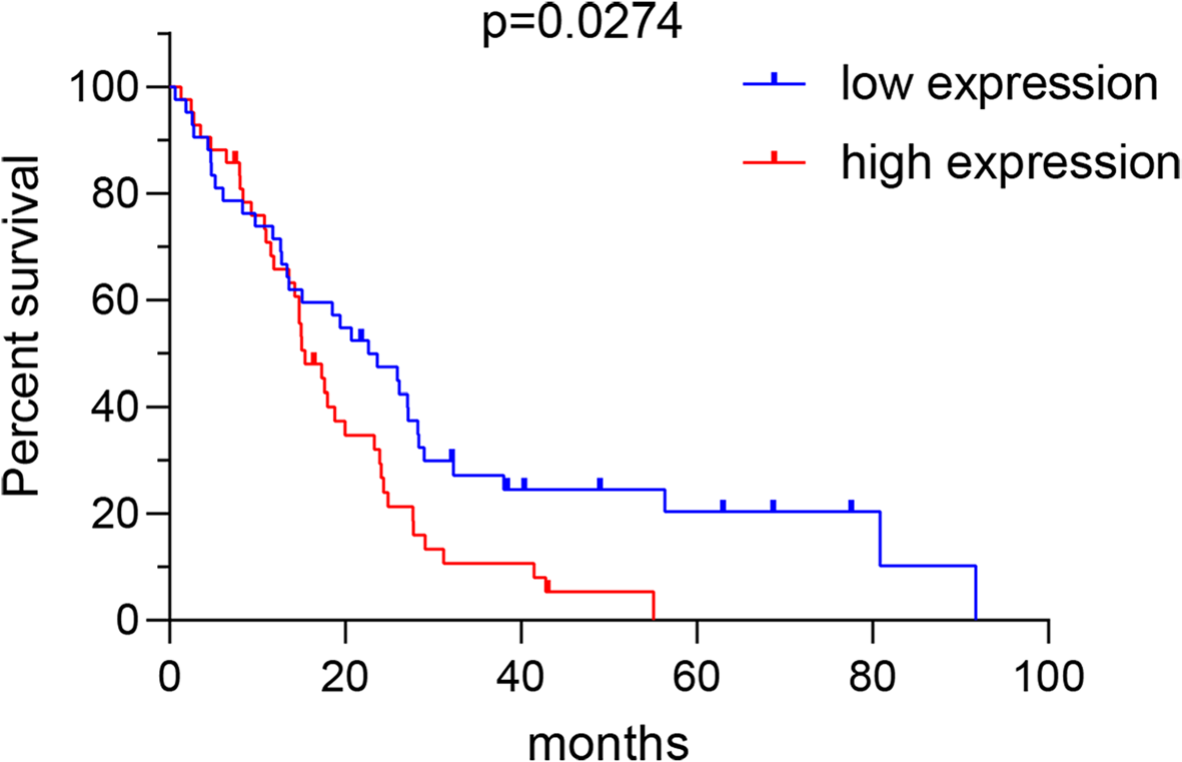
The relationship between NPM2 and the prognosis of patients with MSEO

The RNA sequencing count matrix and the clinical information of the mesothelioma (MSEO) patients were extracted from the Cancer Genome Atlas (TCGA) portal (https://portal.gdc.cancer.gov/). The gene transfer format file (Homo_sapiens.GRCh38.94.chr) was used to obtain the gene symbol for each Ensembl ID in this study. We found a total of 84 pleural MSEO patients, each sample contained NPM2 gene expression information, age, gender, race, survival status, survival time, and pathological type (Table 2). According to the expression level of NPM2, 84 patients were divided into the high-NPM2-expression group and the low-NPM2-expression group. Combined with the survival time and survival status of patients, we used GraphPad Prism8 to draw the survival curve, and the results indicated that the 5-year survival of patients with low-NPM2-expression was significantly higher than that of the high-NPM2-expression patients (Fig. 6).

**Table 2:** The detailed information of 84 MSEO patients from the TCGA database

Variable	Value
Age (years), *n* (%)	
>65	38 (45.2)
≤65	46 (54.8)
Gender, *n* (%)	
Male	69 (82.1)
Female	15 (17.9)
Race, *n* (%)	
Yellow	1 (1.2)
Black	1 (1.2)
White	82 (97.6)
Pathological type, *n* (%)	
Epithelioid mesothelioma	58 (69.0)
Biphasic mesothelioma	21 (25.0)
No classification	5 (6.0)

## Summary

NPM2 is a molecular chaperone that binds histones and plays an important role in chromatin remodeling, genome stability, ribosome biogenesis, DNA duplication, and transcriptional regulation [3, 15–19]. Compared with other members of the NPM family, NPM2 lacks the typical acidic region (NPM2-A1), which may cause that the N-terminal core region cannot directly bind histones. However, the NPM2 C-terminal tail contains a large number of acidic amino acids (NPM2-A2), which can bind to histones and form large complexes. NPM2-A2 plays a crucial role in the binding of NPM2 to histones [4].

NPM2 can facilitate the formation of histone deacetylation [20]. Histone acetylation and deacetylation are important epigenetics functions. They play a regulatory role in active transcription and inhibition of histone and a key role in the development and progression of malignant tumors. Our group established an animal model of MPM and carried out apatinib drug intervention research [21, 22]. It was found that NPM2 expression was significantly downregulated in the apatinib group and the control group by RNA sequencing (RNA-Seq) and quantitative real-time polymerase chain reaction (q-PCR, unpublished data from the authors’ group). Hence, NPM2 may promote histone deacetylation and inhibit the related-gene transcription, thus leading to abnormal proliferation, invasion, and metastasis of MPM. However, the relevant mechanisms still need further in-depth research.

At present, the research on the NPM family mainly focuses on NPM1, and there is a lack of related reports on NPM2. Only a few experiments have shown that the expression or deletion of NPM2 is related to the occurrence, development, prognosis, and outcome of tumors [23–26]. Therefore, the exploration of NPM2 may bring new breakthroughs in the field of MPM diagnosis and treatment.

## Data Availability

Data are available on request from the authors.

## Abbreviations

NPM2: Nucleoplasmin2
MPM: Malignant peritoneal mesothelioma
WHO: World Health Organization
RNA-Seq: RNA sequencing
q-PCR: Quantitative real-time polymerase chain reaction
NPM: Nucleoplasmin
NLS: Nuclear localization signal
MSEO: Mesothelioma

## References

[1] Moolgavkar SH, Meza R, Turim J (2009). Pleural and peritoneal mesotheliomas in SEER: age effects and temporal trends, 1973-2005. Cancer Causes Control..

[2] Travis WD, Brambilla E, Burke AP, Marx A, Nicholson AG (2015). Introduction to the 2015 World Health Organization classification of tumors of the lung, pleura, thymus, and heart. J Thorac Oncol..

[3] Frehlick LJ, Eirin-Lopez JM, Ausio J (2007). New insights into the nucleophosmin/nucleoplasmin family of nuclear chaperones. Bioessays..

[4] Platonova O, Akey IV, Head JF, Akey CW (2011). Crystal structure and function of human nucleoplasmin (npm2): a histone chaperone in oocytes and embryos. Biochemistry..

[5] Boisvert RA, Rego MA, Azzinaro PA, Mauro M, Howlett NG (2013). Coordinate nuclear targeting of the FANCD2 and FANCI proteins via a FANCD2 nuclear localization signal. PLoS One..

[6] Kim HJ, Won HH, Park KJ, Hong SH, Ki CS, Cho SS, Venselaar H, Vriend G, Kim JW (2013). SNP linkage analysis and whole exome sequencing identify a novel POU4F3 mutation in autosomal dominant late-onset nonsyndromic hearing loss (DFNA15). PLoS One..

[7] Nomura T, Watanabe S, Kaneko K, Yamanaka K, Nukina N, Furukawa Y (2014). Intranuclear aggregation of mutant FUS/TLS as a molecular pathomechanism of amyotrophic lateral sclerosis. J Biol Chem..

[8] Paciorkowski AR, Weisenberg J, Kelley JB, Spencer A, Tuttle E, Ghoneim D, Thio LL, Christian SL, Dobyns WB, Paschal BM (2014). Autosomal recessive mutations in nuclear transport factor KPNA7 are associated with infantile spasms and cerebellar malformation. Eur J Hum Genet..

[9] Radivojac P, Iakoucheva LM, Oldfield CJ, Obradovic Z, Uversky VN, Dunker AK (2007). Intrinsic disorder and functional proteomics. Biophys J..

[10] Babu MM, Kriwacki RW, Pappu RV (2012). Versatility from protein disorder. Science..

[11] Dutta S, Akey IV, Dingwall C, Hartman KL, Laue T, Nolte RT, Head JF, Akey CW (2001). The crystal structure of nucleoplasmin-core: implications for histone binding and nucleosome assembly. Mol Cell..

[12] Namboodiri VM, Dutta S, Akey IV, Head JF, Akey CW (2003). The crystal structure of Drosophila NLP-core provides insight into pentamer formation and histone binding. Structure..

[13] Namboodiri VM, Akey IV, Schmidt-Zachmann MS, Head JF, Akey CW (2004). The structure and function of Xenopus NO38-core, a histone chaperone in the nucleolus. Structure..

[14] Lee HH, Kim HS, Kang JY, Lee BI, Ha JY, Yoon HJ, Lim SO, Jung G, Suh SW (2007). Crystal structure of human nucleophosmin-core reveals plasticity of the pentamer-pentamer interface. Proteins..

[15] Laskey RA, Honda BM, Mills AD, Finch JT (1978). Nucleosomes are assembled by an acidic protein which binds histones and transfers them to DNA. Nature..

[16] De Koning L, Corpet A, Haber JE, Almouzni G (2007). Histone chaperones: an escort network regulating histone traffic. Nat Struct Mol Biol..

[17] Das C, Tyler JK, Churchill ME (2010). The histone shuffle: histone chaperones in an energetic dance. Trends Biochem Sci..

[18] Avvakumov N, Nourani A, Côté J (2011). Histone chaperones: modulators of chromatin marks. Mol Cell..

[19] Burgess RJ, Zhang Z (2013). Histone chaperones in nucleosome assembly and human disease. Nat Struct Mol Biol..

[20] Burns KH, Viveiros MM, Ren Y, Wang P, DeMayo FJ, Frail DE, Eppig JJ, Matzuk MM (2003). Roles of NPM2 in chromatin and nucleolar organization in oocytes and embryos. Science..

[21] Yang ZR, Chen ZG, Du XM, Li Y (2020). Apatinib mesylate inhibits the proliferation and metastasis of epithelioid malignant peritoneal mesothelioma in vitro and in vivo. Front Oncol..

[22] Yang ZR, Chen ZG, Ji ZH, Lin YL, Zhang J, Ma R, Li Z, Jiang X, Chen Q, Du XM, Li Y (2021). Establishment and histopathological study of patient-derived xenograft models and primary cell lines of epithelioid malignant peritoneal mesothelioma. Exp Anim..

[23] Koga Y, Pelizzola M, Cheng E, Krauthammer M, Sznol M, Ariyan S, Narayan D, Molinaro AM, Halaban R, Weissman SM (2009). Genome-wide screen of promoter methylation identifies novel markers in melanoma. Genome Res..

[24] Fujiwara S, Nagai H, Jimbo H, Jimbo N, Tanaka T, Inoie M, Nishigori C (2019). Gene expression and methylation analysis in melanomas and melanocytes from the same patient: loss of NPM2 expression is a potential immunohistochemical marker for melanoma. Front Oncol..

[25] Li X, Yang X, Fan Y, Cheng Y, Dong Y, Zhou J, Wang Z, Li X, Wang J (2021). A ten-gene methylation signature as a novel biomarker for improving prediction of prognosis and indicating gene targets in endometrial cancer. Genomics..

[26] Yao L, Cong R, Ji C, Zhou X, Luan J, Meng X, Song N (2021). RNA-binding proteins play an important role in the prognosis of patients with testicular germ cell tumor. Front Genet..

